# Dataset for material logistics on construction sites

**DOI:** 10.1016/j.dib.2018.08.194

**Published:** 2018-09-01

**Authors:** Patience F. Tunji-Olayeni, Adedeji O. Afolabi, Emmanuel E. Eshofonie, Beatrice A. Ayim

**Affiliations:** a Covenant University, Ota, Nigeria; b Big Torch Ventures, Nigeria

**Keywords:** Construction industry, Logistics management, Material management

## Abstract

Data in this article describes logistics management on construction sites in Abuja, Nigeria. Data was elicited from 55 construction professionals comprising of Architects, Builders, Civil Engineers, Project Managers and Quantity Surveyors. The Data set in this study consists of responses on: factors affecting material purchase on construction sites, factors affecting accuracy of material delivery, challenges encountered during material delivery, benefits of material delivery on construction sites and methods of forecasting material demand on construction sites. This article provides insight into logistics management on construction sites in Nigeria and it can be a useful guide for similar research in other contexts.

**Specifications table**

**Table:** 

Subject area	*Construction*
More specific subject area	*Material Logistics*
Type of data	*Tables and Figures*
How data was acquired	*Field Survey*
Data format	*Raw*
Experimental factors	*Random Sampling*
Experimental features	*Descriptive statistics*
Data source location	*Abuja, Nigeria*
Data accessibility	*Data is included*

**Value of the data**

• The data provides insight into the significant factors affecting material purchase on construction sites
• From the data, factors affecting accuracy of material delivery on construction sites can be obtained
• The data presents critical factors to be considered in choosing a material handling equipment on construction sites.
• From the data, the challenges associated with material logistics on construction sites are identified.
• The data in this article can be modified for use in other context.

## 1 Data

Data for this article was solicited from construction professionals in Abuja, Nigeria.

**Fig. 1:**
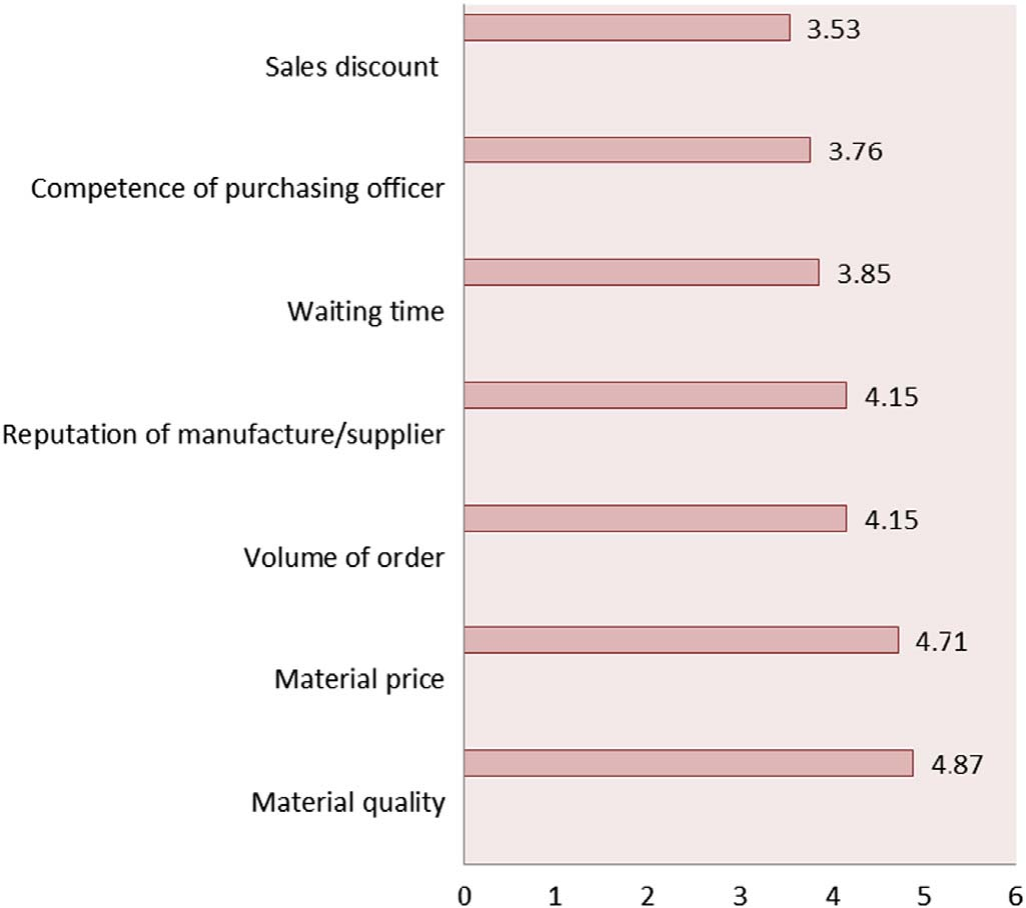
Factors affecting material purchase.

Fig. 1 presents the mean values of the factors affecting material purchase on construction sites. The factors are: material quality (4.87), material price (4.71), volume of order (4.15), reputation of manufacturer (4.15), waiting time (3.85), competence of purchasing officer (3.76) and sales discount (3.53).

**Fig. 2:**
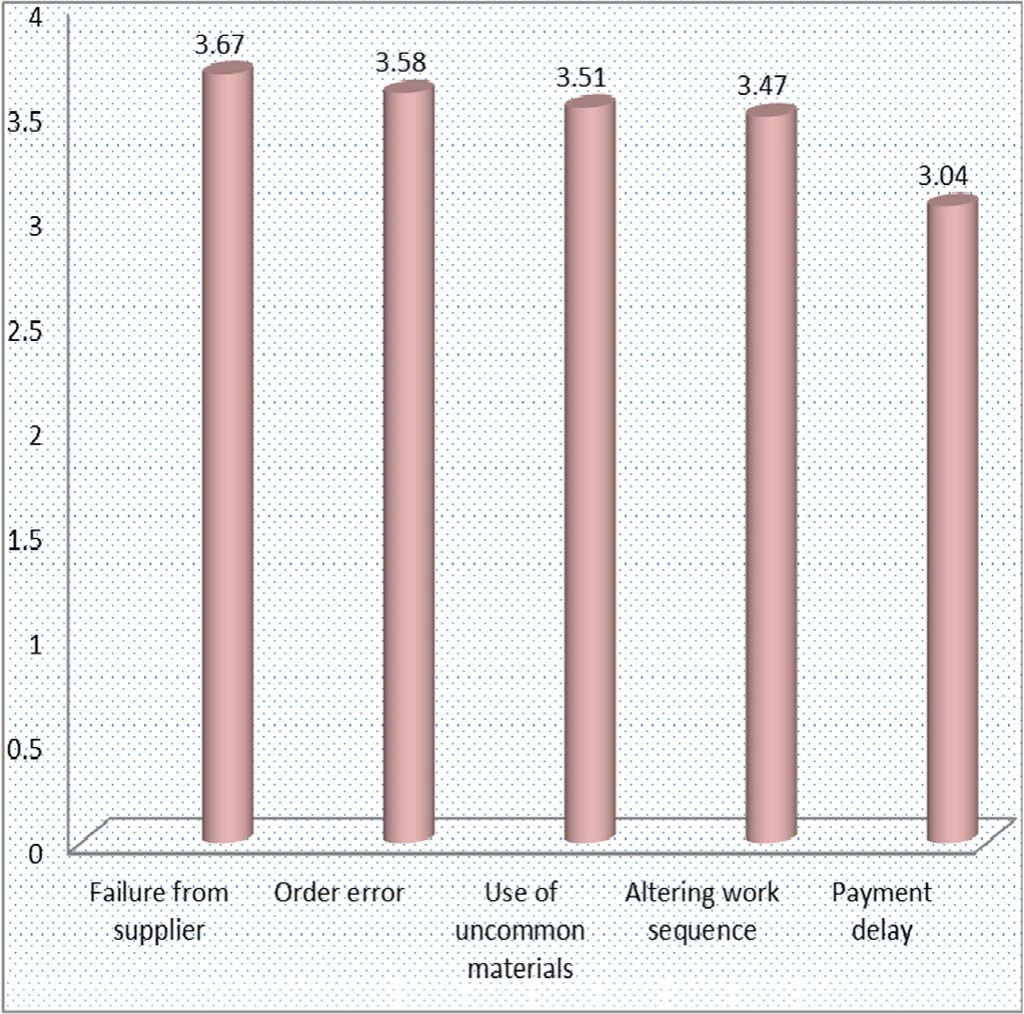
Factors affecting accuracy of material delivery.

Fig. 2 highlights the mean of the factors affecting accuracy of material delivery on construction sites. These factors include: failure from supplier (3.67), order error (3.58), use of uncommon materials (3.51), altering work sequence (3.47) and payment delay (3.04).

**Fig. 3:**
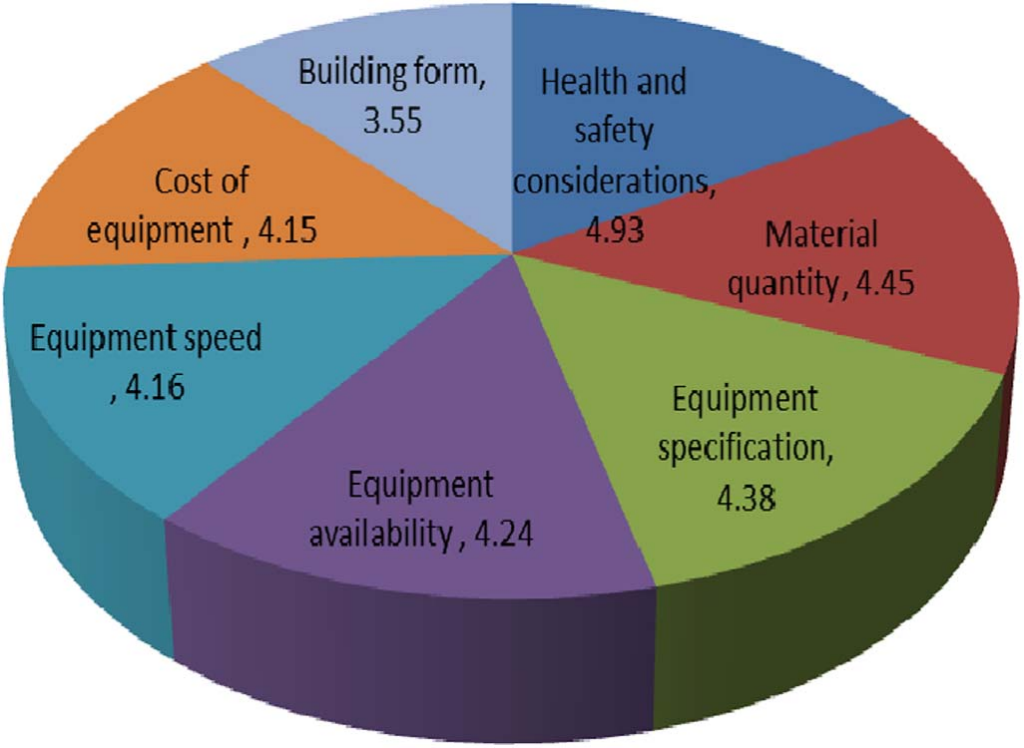
Factors affecting choice of material handling equipment.

Fig. 3 provides the analysis of factors affecting material handling equipment including health and safety considerations (4.93), material quantity (4.45), equipment specification (4.38), equipment availability (4.24), equipment speed (4.16), cost of equipment (4.150) and building form (3.55).

Table 1 shows the challenges associated with material logistics on construction sites. The challenges are: transportation (4.45), inadequate storage (4.18), delay in material delivery (4.13), supply of low quality material (4.07), poor coordination (4.02), inability to forecast activity period (3.91), inaccuracies in material delivery (3.67) and increase in waiting time (3.62).

**Table 1:** Challenges associated with material logistics on construction sites.

Challenge	N	Minimum	Maximum	Mean
Transportation	55	2	5	4.45
Inadequate storage on site	55	2	5	4.18
Delay in material and component delivery	55	1	5	4.13
Supply of low quality material	55	1	5	4.07
Poor coordination among material planning team	55	1	5	4.02
Inability to forecast activity period with accuracy	55	1	5	3.91
Inaccuracies in material delivery	55	1	5	3.67
Increase waiting time between activities	55	1	5	3.62

**Fig. 4:**
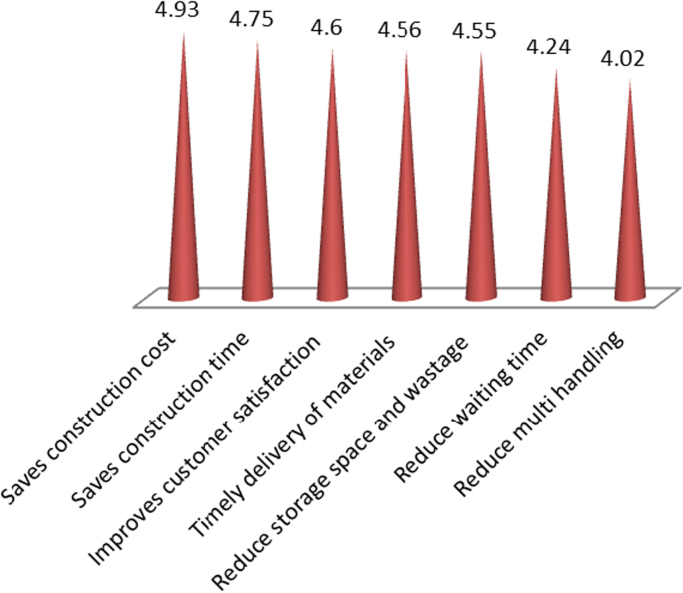
Benefits of material logistics on construction sites.

In Fig. 4, the benefits of material logistics are provided and include: saves construction time (4.93), saves construction cost (4.75), improves customer satisfaction (4.60), timely delivery of materials (4.56), reduce storage space (4.55), reduce waiting time (4.24) and reduce multi handling (4.02).

**Fig. 5:**
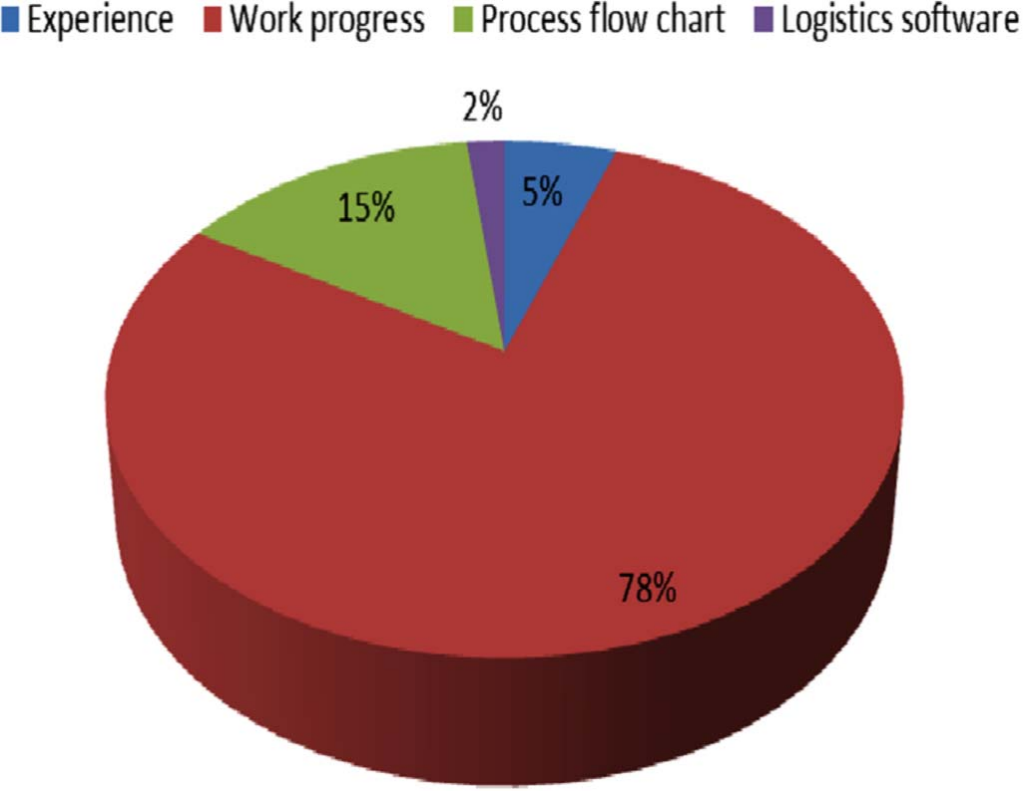
Method of forecasting material demand on construction sites.

Fig. 5, shows the common methods of forecasting material demand in construction sites, which are: work progress (78%), process flow chart (15%), experience (5%) and logistics software (2%).

## 2 Experimental design, materials and methods

The data in this article was generated by means of a cross sectional survey of construction professionals in Abuja, Nigeria. Previous researchers [1], [2], [3], [4], [5], [6], [7], [8] used similar approach to obtain empirical data from respondents. The questionnaire was adapted from similar previous studies [9], [10], [11], [12], [13], [14] and modified. The questionnaire was divided into 6 sections. Section A was used to obtain questions about material purchase on construction sites. Section B covered questions on material handling. Section C had questions that focused on accuracy of material delivery. Section D included questions about the problems encountered in logistics. Section E had questions which probed for the benefits of logistics management. Sections A to E were based on a 5-point likert scale type question with 1= not important, 2 = slightly important, 3 = not sure, 4 = important, 5 = very important. Section F was used to obtain demographic information about the respondents. Seventy questionnaires were distributed to Architects, Builders, Civil Engineers, Project Managers and Quantity Surveyors on a simple random sampling basis. Out of the 70 questionnaires distributed, 55 were returned and found suitable for analysis. Data was analyzed by means of Statistical Package for Social Sciences (SPSS) Version 22.
