# Supplementary material for: Dataset for material logistics on construction sites
Source: Data Brief. 2018 Sep 1;20:1142–7. doi: 10.1016/j.dib.2018.08.194 (PMC6140358; doi:10.1016/j.dib.2018.08.194)
Supplement: Supplementary file 1 — Supplementary material [file mmc1.doc]

**Conflict of Interest**

The authors hereby declare that there is no conflict of interest
