# Supplementary material for: Dataset for material logistics on construction sites
Source: Data Brief. 2018 Sep 1;20:1142–7. doi: 10.1016/j.dib.2018.08.194 (PMC6140358; doi:10.1016/j.dib.2018.08.194)
Supplement: Supplementary file 2 — Supplementary material [file mmc2.doc]

You are invited to take part in a study entitled ‘Material Logistics on Construction Sites’. All information provided will be treated with strict anonymity and used only for academic purposes.

Thank you.

**Material Purchase**

How important are the following factors in determining material purchase on site

(*EI – extremely important, I- important, NS- not sure, SI- slightly important, NI- not important)*

| **S/N** | **Factors** | **EI** | **I** | **NS** | **SI** | **NI** |
| --- | --- | --- | --- | --- | --- | --- |
| 1 | Material price |  |  |  |  |  |
| 2 | Material quality |  |  |  |  |  |
| 3 | Competence of purchasing officer |  |  |  |  |  |
| 4 | Waiting time |  |  |  |  |  |
| 5 | Volume of order |  |  |  |  |  |
| 6 | Reputation of manufacturer/supplier |  |  |  |  |  |
| 7 | Sales discount |  |  |  |  |  |

**Material-Handling Equipment**

How important are the following factors in influencing the choice of material-handling equipment (*EI – extremely important, I- important, NS- not sure, SI- slightly important, NI- not important)*

| **S/N** | **Factors** | **EI** | **I** | **NS** | **SI** | **NI** |
| --- | --- | --- | --- | --- | --- | --- |
| 1 | Material quantity |  |  |  |  |  |
| 2 | Equipment specification |  |  |  |  |  |
| 3 | Equipment speed |  |  |  |  |  |
| 4 | Building form |  |  |  |  |  |
| 5 | Health and safety considerations |  |  |  |  |  |
| 6 | Equipment availability |  |  |  |  |  |
| 7 | Cost of equipment |  |  |  |  |  |

**Accuracy of Material Delivery**

How important are the following factors in determining the accuracy of material delivery

(*EI – extremely important, I- important, NS- not sure, SI- slightly important, NI- not important)*

| **S/N** | **Factors** | **EI** | **I** | **NS** | **SI** | **NI** |
| --- | --- | --- | --- | --- | --- | --- |
| 1 | Payment delay |  |  |  |  |  |
| 2 | Altering work sequence |  |  |  |  |  |
| 3 | Use of uncommon materials |  |  |  |  |  |
| 4 | Failure from supplier |  |  |  |  |  |
| 5 | Order error |  |  |  |  |  |

**Problems in Logistics Management**

How significant are the following problems in logistics management (*ES – extremely significant, S- significant, NS- not sure, SS- slightly significant, NS- not significant)*

| **S/N** | **Factors** | **ES** | **S** | **NS** | **SS** | **NS** |
| --- | --- | --- | --- | --- | --- | --- |
| 1 | Delay in materials and components delivery |  |  |  |  |  |
| 2 | Inability to forecast activity period with accuracy |  |  |  |  |  |
| 3 | Poor coordination of production planning team |  |  |  |  |  |
| 4 | Inaccuracies in material delivery |  |  |  |  |  |
| 5 | Transportation |  |  |  |  |  |
| 6 | Inadequate storage on site |  |  |  |  |  |
| 7 | Increase waiting time between activities |  |  |  |  |  |
| 8 | Supply of low quality material |  |  |  |  |  |

**Advantages of Logistics Management**

How important are the following advantages of Logistics Management in construction firms

(*EI – extremely important, I- important, NS- not sure, SI- slightly important, NI- not important)*

| **S/N** | **Factors** | **EI** | **I** | **NS** | **SI** | **NI** |
| --- | --- | --- | --- | --- | --- | --- |
| 1 | Saves cost |  |  |  |  |  |
| 2 | Saves time of construction work |  |  |  |  |  |
| 3 | Reduce waiting time |  |  |  |  |  |
| 4 | Timely delivery of material |  |  |  |  |  |
| 5 | Reduce storage space and wastage |  |  |  |  |  |
| 6 | Reduce multi-handling |  |  |  |  |  |
| 7 | Improves customer satisfaction |  |  |  |  |  |

Name of organization (optional)………………………………………………………

**Please tick as appropriate**

Who handles material logistics in your firm? Purchase department….Logistics officer….

Project Manager….Owner….

Which is the most frequent method used in forecasting material demand in your organisation?

Experience…Based on work progress….Process flow chart….Logistics software…..

Turnover in Billions of Naira (last year) Below 0.20 Billion….. 0.21-0.50 Billion….0.51-070 Billion….. 0.71-1Billion…..Above 1 Billion…….

Total number of employees: Below 50……51-100 ……101-150 ….151-200….. Above 200….
